# The revised version 2018 of the nationwide web-based registry system for kidney diseases in Japan: Japan Renal Biopsy Registry and Japan Kidney Disease Registry

**DOI:** 10.1007/s10157-020-01932-6

**Published:** 2020-08-06

**Authors:** Takaya Ozeki, Shoichi Maruyama, Michio Nagata, Akira Shimizu, Hitoshi Sugiyama, Hiroshi Sato, Hitoshi Yokoyama, the Committee for Renal Biopsy and Disease Registry of the Japanese Society of Nephrology

**Affiliations:** 1 Department of Nephrology, Nagoya University Graduate School of Medicine, Nagoya, Japan; 2 Faculty of Medicine, Kidney and Vascular Pathology, University of Tsukuba, Tsukuba, Japan; 3 Department of Analytic Human Pathology, Nippon Medical School, Tokyo, Japan; 4 Department of Human Resource Development of Dialysis Therapy for Kidney Disease, Okayama University Graduate School of Medicine, Dentistry and Pharmaceutical Sciences, Okayama, Japan; 5 Department of Internal Medicine, Sendai Hospital of East Japan Railway Company, Sendai, Japan; 6 Department of Nephrology, Kanazawa Medical University School of Medicine, Uchinada, Japan

**Keywords:** Renal biopsy, Pathology, Registry

## Abstract

**Background:**

The Japan Renal Biopsy Registry (J-RBR), the first nation-wide registry of renal biopsies in Japan, was established in 2007, and expanded to include non-biopsy cases as the Japan Kidney Disease Registry (J-KDR) in 2009. The J-RBR/J-KDR is one of the biggest registries for kidney diseases. It has revealed the prevalence and distribution of kidney diseases in Japan. This registry system was meant to be revised after 10 years.

**Methods:**

In 2017, the Committees of the Japanese Society of Nephrology started a project for the revision of the J-RBR/J-KDR. The revised system was designed in such a way that the diagnoses of the patients could be selected from the Diagnosis Panel, a list covering almost all known kidney diseases, and focusing on their pathogenesis rather than morphological classification. The Diagnosis Panel consists of 22 categories (18 glomerular, 1 tubulointerstitial, 1 congenital/genetical, 1 transplant related, and 1 other) and includes 123 diagnostic names. The items for clinical diagnosis and laboratory data were also renewed, with the addition of the information on immunosuppressive treatment.

**Results:**

The revised version of J-RBR/J-KDR came into use in January 2018. The number of cases registered under the revised system was 2748 in the first year. The total number of cases has reached to 43,813 since 2007.

**Conclusion:**

The revised version 2018 J-RBR/J-KDR system attempts to cover all kidney diseases by focusing on their pathogenesis. It will be a new platform for the standardized registration of kidney biopsy cases that provides more systemized data of higher quality.

**Electronic supplementary material:**

The online version of this article (10.1007/s10157-020-01932-6) contains supplementary material, which is available to authorized users.

## Introduction

The Japan Renal Biopsy Registry (J-RBR), the first nation-wide web-based registry of renal biopsies in Japan, was established in 2007 [1]. The renal biopsy is the gold standard for the classification and diagnosis of kidney diseases, and it provides essential information for managing the condition [2,3]. It can also provide us with information on the incidence and distribution of kidney diseases. From the 1980s, the results of renal biopsy registry studies have been reported from all over the world [4–7].

In 2009, the Japan Kidney Disease Registry (J-KDR) which includes non-biopsy cases in addition to those registered in the J-RBR was started [8]. Thereafter, the kidney diseases that do not require renal biopsy, such as polycystic kidney disease and congenital anomalies of the kidney and urinary tract (CAKUT), could also be registered. As of December 2017, 143 nephrology centers have joined the J-RBR/J-KDR, which includes 40,369 patients in total: 37,215 biopsy cases and 3154 non-biopsy cases [9]. The cross-sectional data from the J-RBR/J-KDR have revealed the demographics of kidney diseases in Japan [10–18] and provided fundamental knowledge for ancillary studies [19–24].

In the original J-RBR/J-KDR 2007 system, diagnosis of the patients consists of three components: (i) a clinical diagnosis, (ii) a histological diagnosis by pathogenesis, and (iii) a histological diagnosis by histopathology (Online Resource 1) [1]. It followed the classification of glomerular diseases that was originally proposed in the 1980s by the World Health Organization (WHO) and revised in the 1990s [25,26]. This classification describes histopathological patterns of glomerular injury but does not encompass its etiology. Recently, Sethi et al. suggested a pathogenesis-based classification for glomerulonephritis [27], however, that did not include major proteinuric glomerular diseases, such as minimal change disease (MCD), focal segmental glomerulosclerosis (FSGS), membranous nephropathy (MN), and tubulointerstitial diseases. Therefore, it was necessary to establish a comprehensive classification, which covers all categories of biopsy-proven kidney diseases based on their pathogenesis.

The Committee for Renal Biopsy and Disease Registry of the Japanese Society of Nephrology revised the J-RBR/J-KDR system to establish a more practical histopathological grouping and classification of the kidney diseases.

## Materials and methods

### Participants and data collection

**Fig. 1:**
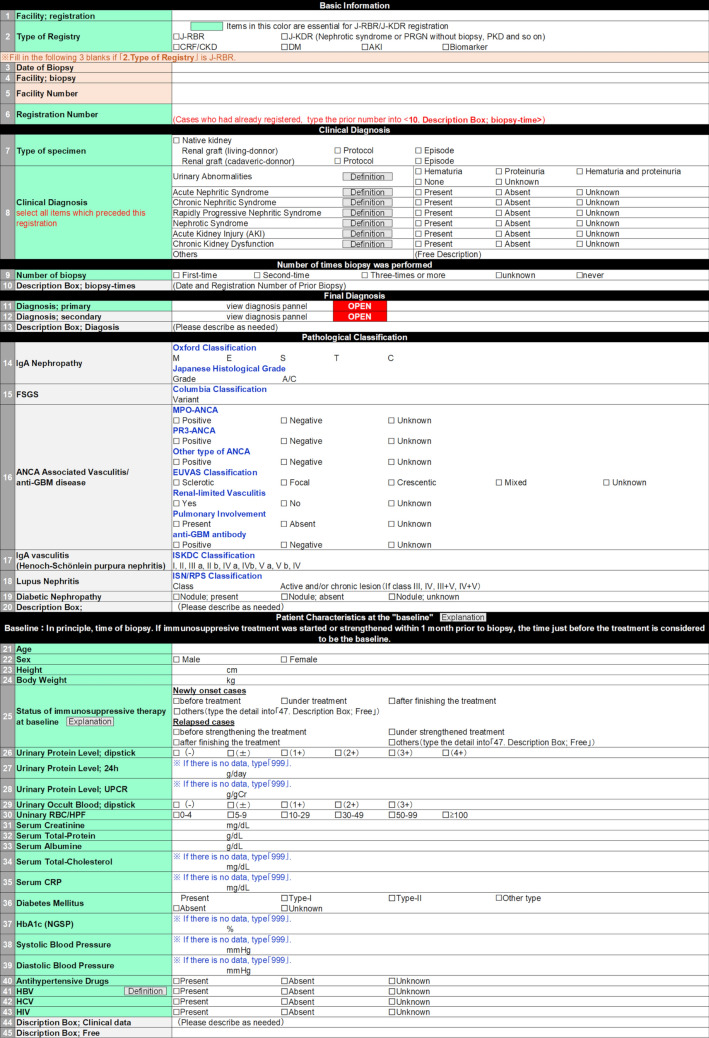
The main page for registration with J-RBR/J-KDR. Definitions: Urinary Abnormalities, hematuria and/or proteinuria observed prior to registration; Acute Nephritic Syndrome, A syndrome characterized by abrupt onset of macroscopic hematuria, proteinuria, hypertension, decreased glomerular filtration and retention of sodium and water [26]. Chronic Nephritic Syndrome, slowly developing renal failure accompanied by proteinuria, hematuria, and hypertension [26]. Rapidly Progressive Nephritic Syndrome, rapidly progressing renal failure within several weeks to several months that is associated with urinary findings, such as proteinuria, hematuria, red blood cell casts, and granular casts indicating glomerulonephritis [28]. Nephrotic Syndrome, both massive proteinuria (≥ 3.5 g/day) and hypoalbuminemia (serum albumin ≤ 3.0 g/dL) [29]. Acute Kidney Injury, (1) Increase serum Creatinine ≥ 0.3 mg/dL within 48 h, (2) Increase serum Creatinine ≥ 1.5 times baseline within 7 days, (3) Urine volume < 0.5 mL/kg/h for 6 h [30]. Chronic Kidney Dysfunction, Cases with eGFR < 60 mL/min/1.75m^2^ for more than 3 months [31]. Pulmonary involvement of ANCA-associated vasculitis/anti-GBM disease, abnormality in chest X-ray except infection or chronic obstructive pulmonary disease (COPD), alveolar hemorrhage and interstitial pneumonia. HBV, cases with prior infection or latent infection are considered as “present”. Explanations: *Patient characteristics at the “baseline”, If immunosuppressive treatment was started or strengthened more than 1 month prior to biopsy, the time of biopsy is considered to be the baseline. If immunosuppressive treatment was started or strengthened within 1 month prior to biopsy but the data just before the treatment were not available, the time of biopsy is considered to be the baseline. † Status of immunosuppressive therapy at baseline, Select the status of immunosuppressive treatment at the time of “baseline”. “after finishing the treatment” indicates the status without any immunosuppressive treatment. *J-RBR* Japan Renal Biopsy Registry, *J-KDR* Japan Kidney Disease Registry, *RPGN* rapid progressive glomerulonephritis, *CRF* chronic renal failure, *CKD* chronic kidney disease, *DM* diabetes mellitus, *AKI* acute kidney injury, *FSGS* focal segmental glomerulosclerosis, *ANCA* anti-neutrophil cytoplasmic antibody, *MPO* myeloperoxidase, *PR3* proteinase 3, *EUVAS* the European Vasculitis Study Group, *GBM* glomerular basement membrane, *ISKDC* the International Study of Kidney Disease in Children, *ISN/RPS* the International Society of Neurology and the Renal Pathology Society, *UPCR* urinary protein creatinine ratio, *RBC* red blood cell, *HPF* hyper power field, *CRP* C-reactive protein, *NGSP* the National Glycohemoglobin Standardization Program, *HBV* hepatitis B virus, *HCV* hepatitis C virus, *HIV* human immunodeficiency virus

The J-RBR/J-KDR collects clinical data and pathological diagnoses of patients from the collaborative institutes in Japan. These data are registered via the web page of the J-RBR/J-KDR (Fig. 1) utilizing the system of Internet Data and Information Center for Medical Research (INDICE) in the University Hospital Medical Information Network (UMIN). The essential points of this revision are described below.

### Clinical diagnoses

The clinical diagnoses of the patients are selected from the list shown in Fig. 1, which includes urinary abnormalities, acute nephritic syndrome, chronic nephritic syndrome, rapidly progressive nephritic syndrome, nephrotic syndrome, acute kidney injury (AKI), chronic kidney dysfunction, and others. These clinical diagnoses are defined by current related guidelines or WHO classification [26,28–31]. The revised system of the J-RBR/J-KDR 2018 allows the registration of multiple clinical diagnoses for each case in case of overlapping clinical symptoms e.g., an MCD case demonstrated with acute kidney injury. A new category for urinary abnormalities was added to describe the patients whose biopsy was followed by asymptomatic mild hematuria and/or proteinuria.

### Pathological diagnoses

1. Overview of the diagnosis panel
  The pathological diagnoses for registration are selected from the “Diagnosis Panel” in the web page of J-RBR/J-KDR. Clicking the button of the panel on the web page opens the list of the diagnoses as shown in Table 1. It is the main part of this revision, and constructed based on two principles. First, we tried to cover all the diagnostic names of kidney diseases including the rare ones. The panel contains 22 categories of renal diseases: 18 glomerular, 1 tubulointerstitial, 1 congenital/genetical, 1 transplant related, and 1 other, including 123 diagnostic names. In the registration process, the most appropriate diagnosis should be selected from the panel as principal diagnosis. For complex cases with multiple diagnoses, such as lupus nephritis with findings of diabetic nephropathy, additional panels for secondary diagnosis are set on the web page of the J-RBR/J-KDR.
  Second, the diagnostic names of the kidney diseases will be registered based on their pathogenesis rather than morphology. For example, a patient with MN induced by hepatitis B virus (HBV) infection is registered into the category of infection-related glomerulonephritis and not MN.
  Additional information, such as the name of specific drug that provoked drug-induced secondary MCD, may be entered in the <13. Description Box; Diagnosis> in the main page of the J-RBR/J-KDR registration (Fig. 1). This panel was reviewed by the Japanese Renal Pathology Society.
2. Detailed pathological classification (optional)

**Table 1:** List of the diagnoses in the Diagnosis Panel of the J-RBR/J-KDR

**1. IgA nephropathy**
1) Primary IgA nephropathy
2) Secondary IgA nephropathy
(1) Hepatological disorder*
(2) Others*
**2. Minimal change disease (MCD)**
1) Primary (idiopathic) MCD
2) Secondary MCD
(1) Malignancy*
(2) Drug-induced*
(3) Others*
**3. Focal segmental glomerulosclerosis (FSGS)**
1) Primary (idiopathic) FSGS^a^
2) Secondary FSGS
(1) Familial/genetic*
(2) Obesity
(3) Low birth weight*
(4) Hypertension/arteriosclerosis*
(5) Drug-induced*
(6) Others*
**4. Membranous nephropathy**
1) Primary (idiopathic) Membranous nephropathy
2) Secondary Membranous nephropathy
(1) Malignancy*
(2) Drug-induced*
(3) Infection*^,b^
(4) Others*
**5. Membranoproliferative glomerulonephritis (MPGN)**
1) Primary MPGN^c^
(1) Type I MPGN
(2) Type III MPGN*
2) Secondary MPGN^d^
(1) Secondary MPGN*
(2) Others*
**6. C3 glomerulopathy**
1) Dense deposit disease (DDD)
2) C3 glomerulonephritis
**7. Vasculitis syndrome**^e^
1) ANCA-associated vasculitis
(1) Microscopic polyangiitis (MPA)
(2) Granulomatous polyangiitis (GPA)
(3) Eosinophilic granulomatous polyangiitis (EGPA)
(4) Drug-induced*
(5) Unclassified*
2) Anti-glomerular basement membrane (GBM) disease^f^
3) IgA vasculitis (Henoch-Schönlein purpura nephritis)^g^
4) Polyarteritis nodosa
5) Others*^,h^
**8. Nephropathy associated with connective tissue diseases**
1) Lupus nephritis^i^
2) Sjögren syndrome
(1) Tubulointerstitial nephritis
(2) Others*
3) Rheumatoid arthritis*^,j^
4) Systemic sclerosis
(1) Thrombotic microangiopathy
(2) Others*
5) Others*
**9. Infection related glomerulonephritis**
1) Poststreptococcal acute glomerulonephritis
2) Staphylococcus associated glomerulonephritis*
3) HBV-associated nephropathy
(1) Membranous nephropathy
(2) Others*
4) HCV-associated nephropathy
(1) MPGN
(2) Others*
5) Parvovirus related glomerulonehritis
6) HIV associated nephropathy
7) Others*
**10. Other glomerulonephropathies**
1) IgM nephropathy
2) C1q nephropathy
3) Others*
**11. Hypertension/arteriosclerosis**
1) Nephrosclerosis
(1) Essential hypertension/arteriosclerosis
(2) Malignant hypertension
2) Choresterol crystal embolization
3) Others*^,k^
**12. Thrombotic microangiopathy(TMA)・endothelial injury**
1) Shiga toxin-production E coli hemolytic uremic syndrome (STEC-HUS)
2) Atypical hemolytic uremic syndrome (aHUS)
3) Preeclampsia
4) Drug-induced*
5) Others* ^l^
**13. Diabetic nephropathy**
1) Diabetic nephropathy
**14. Nephropathies with altered lipid metabolism**
1) Lipoprotein glomerulopathy
2) LCAT deficiency
3) Others*
**15. Paraprotein-related kidney disesase**^m^
1) Monoclonal immunoglobulin deposit disease (MIDD)
(1) Light chain deposition disease (LCDD)
(2) Heavy chain deposition disease (HCDD)
(3) Light and heavy chain deposition disease (LHCDD)
2) Proliferative glomerulonephritis with monoclonal IgG deposits (PGNMID)^n^
3) Cast nephropathy*
4) Others*
**16. Cryoglobulinemic vasculitis**
1) Cryoglobulinemic vasculitis^o^
(1) Hematological/lymphoproliferative disorders*
(2) Others/unknown etiology*
**17. Nephropathies with organized deposit**
1) Immunotactoid glomerulopathy
2) Fibrillary glomerulonephritis
3) Fibronectin glomerulopathy
4) Collagenofibrotic nephropathy
5) Others*
**18. Renal amyloidosis**
1) AA amyloidosis*
2) AL amyloidosis*
3) Other type of amyloidosis*^,p^
**19. Congenital/genetic**
1) Congenital nephrotic syndrome^q^
2) Alport syndrome
3) Thin basement membrane disease
4) Fabry disease
5) Renal disease associated with mitochondrial cytopathy
6) Autosomal dominant tubulointerstitial kidney disease (ADTKD): including medullary cystic kidney disease (MCKD)
7) Nephronophthisis/nephronophthisis-associated ciliopathies
8) Polycystic kidney disease
(1) Autosomal dominant polycystic kidney disease (ADPKD)
(2) Autosomal recessive polycystic kidney disease (ARPKD)
(3) Others
9) Congenital anomalies of the kidney and urinary tract (CAKUT)
(1) Syndromic CAKUT
(2) Non-syndromic CAKUT
10) Nail-patella syndrome/LMX1B associated nephropathy
11) Others*
**20. Tubulointerstitial nephropathies**
1) Tubulointerstitial nephritis
(1) Drug-induced*
(2) IgG4-related kidney disease
(3) Sarcoidosis
(4) Tubulointerstitial nephritis and uveitis (TINU) syndrome
(5) Others*^,r^
(6) Unknown
2) Acute tubular necrosis
3) Others*
**21. Transplant kidney**
1) Transplant rejection
(1) Hyperacute rejection
(2) Acute rejection
① Acute antibody mediated rejection
② Acute T-cell mediated rejection
(3) Chronic rejection
① Chronic antibody mediated rejection
② Chronic T-cell mediated rejection
(4) Others*
2) Drug-induced graft injury
(1) Calcineurin inhibitor induced nephropathy
(2) Others*
3) Transplant related infection
(1) BK virus
(2) Adenovirus
(3) Epstein–Barr virus^s^
(4) Cytomegalovirus
(5) Others*
4) Post-transplant lymphoproliferative disorders (PTLD)
5) No specific findings
6) Others*
**22. Others**
1) No specific abnormalities
2) Others*
3) Undiagnosable*

*J-RBR* Japan Renal Biopsy Registry, *J-KDR* Japan Kidney Disease Registry, *ANCA* anti-neutrophil cytoplasmic antibody, *HBV* hepatitis B virus, *HCV* hepatitis C virus, *HIV* human immunodeficiency virus, *LCAT* lecithin-cholesterol acyltransferase

*Describe the details in the < 13. Description Box; Diagnosis > in the main page of the registration for the J-RBR/J-KDR (Fig. 1).

^a^Describe the Columbia classification in < 15. FSGS > in the main web page (Fig. 1)

^b^Cases related to HBV or HCV should be registered in **9. Infection related glomerulonephritis**

^c^MPGN type II (DDD) should be registered in **6. C3 glomerulopathy**

^d^Cases related to HBV or HCV should be registered in **9. Infection related glomerulonephritis**

^e^If the patient has other underlying diseases, e.g., systemic sclerosis, the details should be described in < 13. Description Box; Diagnosis > in the main web page (Fig. 1). ANCA-negative ANCA associated vasculitis should also be categorized into MPA, GPA or EGPA

^f^Describe the data about antibodies/pathological classifications into < 16. ANCA Associated Vasculitis/anti-GBM disease > in the main web page (Fig. 1)

^g^Describe the pathological classification into < 17. Henoch-Schönlein purpura nephritis > in the main web page (Fig. 1)

^h^Cases with cryoglobulinemic vasculitis should be registered in **16. Cryoglobulinemic vasculitis**

^i^Describe the pathological classification in < 18. Lupus Nephritis > in the main web page (Fig. 1)

^j^Cases with membranous nephropathy or amyloidosis should be registered into their distinct categories

^k^Cases with FSGS lesion should be categorized into secondary FSGS

^l^TMA associated with systemic sclerosis should be categorized into **8. Nephropathy associated with connective tissue diseases**

^m^Cases with amyloid deposition should be registered in **18. Renal amyloidosis**

^n^Describe the subtype of immunoglobulin in < 13.Description Box; Diagnosis > in the main web page (Fig. 1)

^o^Cases with the infectious etiologies, such as HCV, should be categorized into **9. Infection related glomerulonephritis**. Cases with the etiologies of connective tissue diseases such as SLE, should be categorized into **8. Nephropathy associated with connective tissue diseases**. In these cases, describe the information on cryoglobulinemia in < 13. Description Box; Diagnosis > in the main web page (Fig. 1)

^p^Cases which do not have any information about AA/AL should also be registered to **3) Other type of amyloidosis** in **18. Renal amyloidosis**

^q^Describe pathological diagnosis in < 13.Description Box; Diagnosis > in the main web page (Fig. 1) if available

^r^Cases who associated with any infection should be registered to **7) Others** in **9. Infection related glomerulonephritis**

^s^Cases with Epstein-Barr virus related PTLD should be registered in PTLD

In this revision, we added more items for detailed pathological classification of several glomerular diseases, such as the Oxford Classification [32–34] and Japanese Histological Grade [35] for IgA nephropathy, the Columbia Classification for FSGS [36], the International Society of Neurology and the Renal Pathology Society (ISN/RPS) classification for Lupus nephritis [37], the International Study of Kidney Disease in Children (ISKDC) classification for Henoch-Schönlein purpura nephritis [38], and the European Vasculitis Study Group (EUVAS) classification for ANCA-associated vasculitis [39].

### Clinical data

1. Physical measurements and laboratory data
  The revised system collects baseline clinical data (Fig. 1). The baseline is defined in the next section. The clinical variables include patient characteristics and physical measurements (age, sex, height, body weight, and systolic/diastolic blood pressure), comorbidities (hypertension, diabetes, infection of HBV, hepatitis C virus, or human immunodeficiency virus), urinary findings (qualitative testing for urinary protein, occult blood, and red blood cells, and quantitative measurement of urinary protein creatinine ratio and daily proteinuria), and blood test findings (serum creatinine, total protein, albumin, total cholesterol, CRP, and HbA1c). Estimated glomerular filtration rate (eGFR) is calculated from the patient’s age, sex, and serum creatinine level using the equations for Japanese children [40] and adults [41] at the time of analysis.
2. Treatment status of the “baseline”

In patients who received immunosuppressive treatment before biopsy, laboratory data at biopsy may be modified by the treatment. Information on the treatment status including whether the case is a new onset one or relapsed one will also be collected. The “baselines” for collecting clinical data are defined as follows (Fig. 1). Regarding the cases in which immunosuppressive treatment started or strengthened within 1 month prior to biopsy, the time just before the initiation of treatment is considered to be the baseline. In the cases in which immunosuppressive treatment started or strengthened more than 1 month prior to biopsy, the time of biopsy is considered as the baseline. In the cases in which immunosuppressive treatment started or strengthened within 1 month prior to biopsy but the data just before the treatment were not available, the time of biopsy is considered to be the baseline.

## Results

The revised system of J-RBR/J-KDR came into use in January 2018. As of December 2018, the number of cases registered under the revised system had reached 2748 in the first year from 146 facilities. The total number of the cases registered in the J-RBR/J-KDR had reached 43,813 from the beginning of J-RBR in 2007.

## Discussion

The J-RBR/J-KDR is a nationwide web-based registry in Japan, which has been conducted for more than 10 years, and it is one of the biggest registries for patients with kidney diseases including biopsy cases. We revised its registration system in 2018 with several strengths.

### Clinical diagnoses that can represent the clinical status of the patients

The clinical diagnoses in the revised system enable to describe the clinical status of the patients more accurately based on the following modifications. First, the revised J-RBR/J-KDR allows the selection of all clinical diagnoses that are appropriate for the patients from the 8 listed items. Although the clinical indications for kidney biopsy vary [6], we sometimes experience patients who have multiple clinical symptoms that lead to biopsy. For example, 20–30% of the patients with MCD were reported to have demonstrated AKI [42]. If only one clinical diagnosis is registered in such cases, both the clinical diagnoses, nephrotic syndrome and AKI, could be underestimated. Second, the items indicating abnormalities in urinalysis were added. In Japan, a nationwide annual health examination program including urinalysis screening for all community residents has been going on for over 40 years [43]. It enables early detection of urine abnormalities and early referral to a nephrologist, and it is possible that a substantial number of the patients have undergone renal biopsy due to asymptomatic hematuria and/or proteinuria. Therefore, the revised J-RBR/J-KDR 2018 can describe clinical features of such patients.

### Classification of kidney diseases and the structure of their registry system

For kidney diseases, the classification system should meet the following requirements: (a) clinically significant, useful, and therapeutically relevant, (b) based on pathogenesis within current knowledge, (c) easy to use and morphologically reproducible and (d) able to provide the information for prognosis [44]. Although the terminology of kidney diseases has been mainly based upon morphology, the pathogenesis of kidney diseases has been gradually revealed in recent decades. For example, membranous proliferative glomerulonephritis (MPGN) has been recognized as a pattern of glomerular injury and indicates a grouping of the patients who share common light microscopic findings. Nowadays, the cases with dysregulation of the alternative pathway of complement in MPGN are subdivided into C3 glomerulopathy [45]. Considering these advances, a classification based on etiology and pathogenesis is more desirable [44].

In addition, the value of a registry depends on its data quality, clarity of registration method, and ease of using the collected data [46]. The WHO classification [25,26], which was the fundamental concept behind the original J-RBR/J-KDR 2007 system, embodies the idea that the pathology of kidney diseases should be comprehensively interpreted with the integration of clinical, histopathological, and pathognomonic diagnoses. However, this classification was basically weighted in favor of morphology, not pathogenesis. Further, this complicating method made difficulty in data aggregation and not all diagnoses are suitable for being expressed by this method (e.g., MCD or diabetic nephropathy). In this revised system, the most appropriate diagnosis for the patients can be selected from the diagnosis panel, and this simple method will provide high reproducibility in registration.

### Clinical data collection with the information of treatment status

Most of registries for kidney diseases collect the clinical data at the time of biopsy. However, it is difficult to interpret the laboratory data of patients without information on the treatment status. For example, we cannot distinguish the patients who demonstrated non-nephrotic range proteinuria from those who showed the improvement in nephrotic syndrome as a result of immunosuppressive treatment from their laboratory data alone. The revised J-RBR/J-KDR 2018 collects the information whether the laboratory data were collected before or after starting the immunosuppressive treatment. In this revision, based on these status, different “baselines” are defined for each case and it will provide more reliable data to describe the laboratory features of kidney diseases.

### Problems requiring further discussion

The revised J-RBR/J-KDR 2018 system has several points that require further discussion. First, the borderline between the terms, “Primary (idiopathic)” and “Secondary,” in the glomerular disease is ambiguous. For example, primary MCD and FSGS are considered as a spectrum of diseases that are provoked by humoral permeability factors [47], while secondary cases indicate the presence of an identifiable etiology [48,49]. However, the identities of the permeability factors for MCD/FSGS are yet to be revealed [50]. For MN, the antibodies against potent etiological factors, such as the phospholipase A2 receptor (PLA2R) [51] and thrombospondin type-1 domain-containing 7A (THSD7A) [52], have been detected. Therefore, the classification based on the terminology of “primary” that indicates an unknown etiology may no longer make sense for patients with MN [53].

Second, this revision does not completely cover the recently reported disorders, such as tubulointerstitial nephritis with IgM-positive plasma cells [54]. Furthermore, in the near future, the newly developed technical approaches in kidney research including multi-omics analysis will probably reveal new pathogenesis for kidney diseases [55,56],therefore, it is necessary to continue the revision of the diagnosis panel with every new result.

## Conclusion and future perspectives

The revised J-RBR/J-KDR 2018 system has attempted to cover the current classification and diagnosis of kidney diseases based on pathogenesis. It can provide data of higher quality on the demographics of kidney diseases and their classifications. In addition, the revised J-RBR/J-KDR 2018 system will be a platform for standardized registration of kidney diseases, and with this platform, we expect to promote international collaborations in research.

## Electronic supplementary material

Below is the link to the electronic supplementary material.

Supplementary file1 (DOCX 57 kb)

Supplementary file2 (DOCX 27 kb)
