# Supplementary material for: The revised version 2018 of the nationwide web-based registry system for kidney diseases in Japan: Japan Renal Biopsy Registry and Japan Kidney Disease Registry
Source: Clin Exp Nephrol. 2020 Aug 6;24(11):1058–68. doi: 10.1007/s10157-020-01932-6 (PMC7524691; doi:10.1007/s10157-020-01932-6)
Supplement: Supplementary file 1 — Supplementary file1 (DOCX 57 kb) [file 10157_2020_1932_MOESM1_ESM.docx]

**Supplementary Table 1. Registration system for diagnoses of the patients in the previous version of the J-RBR/J-KDR**


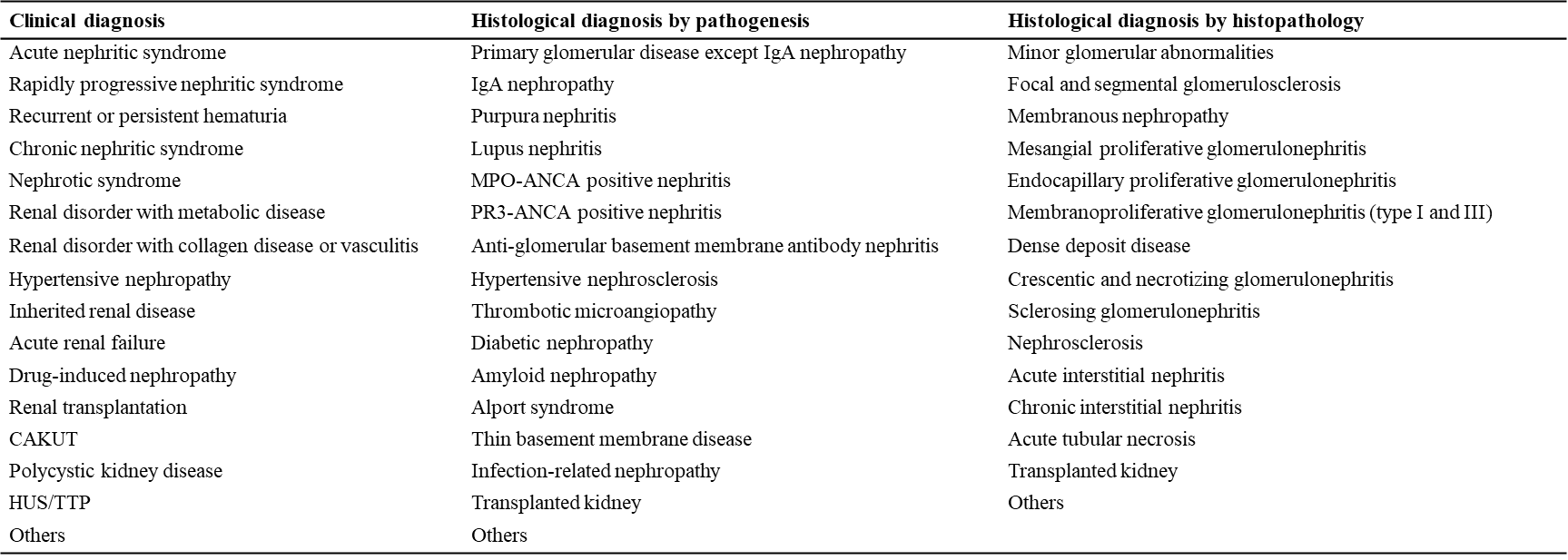


Registration system for the diagnoses of the patients in the previous version of the J-RBR/J-KDR system consisted of three components based on the WHO classification for glomerular diseases: (i) clinical diagnosis, (ii) histological diagnosis by pathogenesis, and (iii) histological diagnosis by histopathology.

**Abbreviations:** J-RBR, Japan Renal Biopsy Registry; J-KDR, Japan Kidney Disease Registry; CAKUT, Congenital anomalies of the kidney and urinary tract; HUS, Hemolytic uremic syndrome; TTP, Thrombotic thrombocytopenic purpura; MPO, myeloperoxidase; ANCA, anti-neutrophil cytoplasmic antibody; PR3, proteinase 3; WHO, World Health Organization
